# Behavioral Profiles of Clinically Referred Children with Intellectual Giftedness

**DOI:** 10.1155/2013/540153

**Published:** 2013-07-10

**Authors:** Fabian Guénolé, Jacqueline Louis, Christian Creveuil, Jean-Marc Baleyte, Claire Montlahuc, Pierre Fourneret, Olivier Revol

**Affiliations:** ^1^CHU de Caen, Service de Psychiatrie de l'Enfant et de l'Adolescent, avenue Clemenceau, 14033 Caen Cedex 9, France; ^2^Hospices Civils de Lyon, Service Hospitalo-Universitaire de Psychiatrie de l'Enfant et de l'Adolescent, Hôpital Femme-Mère-Enfant, 59 boulevard Pinel, 69500 Bron, France; ^3^CHU de Caen, Unité de Biostatistiques et de Recherche Clinique, avenue Clemenceau, 14033 Caen Cedex 9, France; ^4^Université de Normandie, Faculté de Médecine, avenue de la Côte de Nacre, 14032 Caen Cedex 5, France; ^5^Université Claude Bernard Lyon-1, Faculté de Médecine Lyon Est, 8 avenue Rockefeller, 69373 Lyon Cedex 8, France

## Abstract

It is common that intellectually gifted children—that is, children with an IQ ≥ 130—are referred to paediatric or child neuropsychiatry clinics for socio-emotional problems and/or school underachievement or maladjustment. These clinically-referred children with intellectual giftedness are thought to typically display internalizing problems (i.e., self-focused problems reflecting overcontrol of emotion and behavior), and to be more behaviorally impaired when “highly” gifted (IQ ≥ 145) or displaying developmental asynchrony (i.e., a heterogeneous developmental pattern, reflected in a significant verbal-performance discrepancy on IQ tests). We tested all these assumptions in 143 clinically-referred gifted children aged 8 to 12, using Wechsler's intelligence profile and the Child Behavior Checklist. Compared to a normative sample, gifted children displayed increased behavioral problems in the whole symptomatic range. Internalizing problems did not predominate over externalizing ones (i.e., acted-out problems, reflecting undercontrol of emotion and behavior), revealing a symptomatic nature of behavioral syndromes more severe than expected. “Highly gifted” children did not display more behavioral problems than the “low gifted.” Gifted children with a significant verbal-performance discrepancy displayed more externalizing problems and mixed behavioral syndromes than gifted children without such a discrepancy. These results suggest that developmental asynchrony matters when examining emotional and behavioral problems in gifted children.

## 1. Introduction

 Although the whole population of intellectually gifted children—that is, children with an intellectual quotient (IQ) higher or equal to 130, according to the main and most consensual definition [1]—seems not to display increased psychiatric morbidity [2], it is highly common that certain of them are referred to paediatric or child neuropsychiatry clinics for socioemotional problems and/or school underachievement or maladjustment [3–6]. Behavioral and emotional problems typically described in intellectually gifted children are anxiety [7], social withdrawal [8, 9], low self-esteem [10], and excessive perfectionism [7], which all belong to the category of “internalizing” problems [11]. This common observation of internalizing behavioral problems in gifted children without an increased prevalence of internalizing categorical disorders (i.e., anxiety and mood disorders) in the whole gifted population incites to study its psychopathology dimensionally [12] and also to consider its potential heterogeneity [13].

Indeed, it is long considered that, among gifted children, those with higher IQs display increased adjustment problems [14]. Significant difficulties in social adjustment were common for children with the highest IQs in the famous Terman cohort [15], and Hollingworth reported difficulties increasing with IQ regarding socio-emotional and educational adjustment [16, 17]. Hollingworth's work suggested that the most desirable intellectual level for gifted children was an IQ below 145, a higher one representing a risk factor regarding socio-emotional and educational maladjustment [16]. This was subsequently corroborated by Lewis, who found that gifted children with IQs ≥ 145 were more maladjusted than the low gifted [18], and Shaywitz and collegues reported increased behavioral problems in highly gifted children compared to the low gifted [13]. A study in clinically referred gifted children points to the same direction, showing socio-emotional problems increasing with IQ [3].

Another notion frequently mentioned when interpreting socio-emotional and educational maladjustment of gifted children is “developmental asynchrony” [19, 20], a term which designates a problematic pattern of heterogeneities between cognitive, emotional, and psychomotor levels, which is seen in the development of gifted children. Psychometrically, developmental asynchrony may be reflected on Wechsler's IQ tests in the verbal-performance discrepancy [21], which quantifies the cognitive imbalance between abilities in verbal abstraction and concrete nonverbal reasoning [22]. Examination of the verbal-performance discrepancy is the hallmark of Wechsler's intelligence profile analysis, with a value ≥15 being considered as significant and indicative of an abnormal profile [22, 23]. A significant verbal-performance discrepancy (SVPD) is seen in approximately one quarter of gifted children [24]—with a verbal prominence in almost all cases—and some data suggest that it is more frequent in gifted children who are clinically referred [6, 25]. SVPD was found to be associated with social and school maladjustment in gifted children [26], and verbal prominence in clinically referred children with intellectual giftedness was found to be associated with the most serious behavioral symptoms [3].

The purpose of this study was to add to the limited literature related to behavioral profiles of clinically referred children with intellectual giftedness. Our research hypotheses were that (1) they would display increased behavioral problems compared to a normative sample; (2) their behavioral problems would predominate in the internalizing domain; (3) highly gifted children (IQ ≥ 145) would display more behavioral problems than low gifted children (130 ≤ IQ < 145); and (4) gifted children with an SVPD would display more behavioral problems than gifted children without an SVPD.

## 2. Methods

### 2.1. Subjects

The “gifted” group consisted of 144 children, 42 girls (29.2%) and 102 boys (70.8%) aged 8 to 11 (mean: 9.3 ± 1.0 years) and with a full-scale IQ (FSIQ) higher or equal to 130 on the French version of the *Wechsler Intelligence Scale for Children—Third Edition* [22]. They were recruited at the department of child and adolescent psychopathology of the “*Hospices Civils de Lyon*” (France) and through the private practice of four paediatricians in Lyon, where they were referred because of socio-emotional problems and/or school underachievement or maladjustment. Among units, the department of child and adolescent psychopathology of the “*Hospices Civils de Lyon*” includes a reference center for learning disabilities; the four paediatricians were regular correspondents of the department. All children were examined by trained psychiatrists and psychologists, who performed categorical mental disorder diagnoses according to the fourth version revised of the *Diagnostic and Statistical Manual of Mental Disorders* [27]. In parallel, a control group matched one-to-one with the “gifted group” for age and gender was recruited in five primary schools randomly chosen among those of the city of Lyon.

Parents of participating children were asked to complete a document comprising the French version of the Child Behavior Checklist (CBCL) and a form for the collection of sociodemographic data. In accordance with the declaration of Helsinki and with the French law, they all signed informed consent after receiving a full description of the study and explanation of its purpose. Results were collected in an anonymous database, according to the requirements of the French national committee for private freedoms.

### 2.2. The Child Behavior Checklist

The CBCL [28] is a well-established and internationally recognized device for a dimensional assessment of general psychopathology in children and adolescents. It consists of 118 statements about which parents are asked to answer on a 3-point Likert scale how much they apply to their children considering the last 6 months. The CBCL provides a “total score” (TS) for behavioural problems, which can be dichotomized into “internalizing problems” (IP; i.e., self-focused problems, such as feelings of worthlessness or inferiority, dependency, anxiety, excessive sadness, or social withdrawal, which denote overcontrol of behavior and emotion) and “externalizing problems” (EP; i.e., acted-out problems, such as hyperactivity, irritability, rule breaking, or belligerence, which denote undercontrol of behavior and emotion) scores. Based on factor analyses that identified patterns of co occurring items [28], the CBCL also allows individualizing 8 narrow-band dimensional subscores: “withdrawn” (WI), “somatic complaints” (SC), “anxious/depressed” (AD), “social problems” (SP), “thought problems” (TP), “attention problems” (AP), “delinquent behavior” (DB), and “aggressive behavior” (AB).

The French version of the CBCL [29] displays well-validated psychometric properties [30], including discriminant validity between referred and nonreferred children [31, 32] and confirmation of the 8-syndrome model [33].

### 2.3. Data Analysis

Results of one gifted child were removed from analyses because of incorrect filling of the CBCL form; thus, gifted and control groups finally consisted of 143 children.

Sociodemographic (categorical) variables were compared across both groups using chi-square tests; CBCL raw scores and subscores were compared using Student's *t*-tests. 

IP and EP standard T-scores (normalized on the distribution in the control group, with 50 indicating average and every 10 points representing one standard deviation) were compared in the gifted group using Student's *t*-test.

CBCL raw scores and subscores were compared within the gifted group between children with FSIQ comprised between +2 and +3 standard deviations above normal average (“low gifted” children: 130 ≤ FSIQ < 145) and children with FSIQ higher than +3 standard deviation above normal average (“highly gifted” children: FSIQ ≥ 145), using Student's *t*-tests. Proportions of children whose scores exceeded cut-off norms for IP (internalizing syndrome), EP (externalizing syndrome), or both (mixed syndrome) were compared across these two groups using chi-square tests or Fisher's exact tests (depending on validity's condition); 90th percentile of scores and subscores distributions in the normative group were used as scale norms, as it is the recommended cut-off for differentiating cases and noncases in French community samples [30, 34]. The same comparisons were performed between children with and without an SVPD (verbal-performance discrepancy ≥ 15). 

CBCL data were computed within the software *Assessment Data Manager (ADM)* version 7.00 (http://www.aseba.org/); statistical analyses were performed with the software *R* version 2.15.0 (http://www.r-project.org/); the term “significant” denotes statistical differences at the *P* < 0.05 level.

## 3. Results

Sociodemographic characteristics for both groups are listed in Table 1. There was no significant difference for sibling rank, matrimonial situation, and employment of parents. Proportions of parents with high education levels were significantly higher in the gifted group. 

Compared mean CBCL scores and subscores are detailed in Table 2. All results were significantly higher in the gifted group.

Mean IQ results in the gifted group were as follow: FSIQ: 138.6 ± 6.6 (range: 130–160); verbal scale IQ: 137.3 ± 7.3 (range: 121–155), performance scale IQ: 127.6 ± 9.0 (range: 108–155). Among the 143 children, 114 (79.7%) were “low gifted,” and 29 (20.3%) were “highly gifted”; 51 (35.7%) displayed an SVPD, and 92 (64.3%) did not. Among the 51 children with an SVPD, verbal scale IQ predominated in 48 cases (94.1%). No child was diagnosed as suffering from any categorical mental disorder according to DSM-IV-TR.

Mean IP and EP standard T-scores in the gifted group did not significantly differ (59.8 ± 13.8  *versus*  61.2 ± 14.6, resp.).

**Figure 1:**
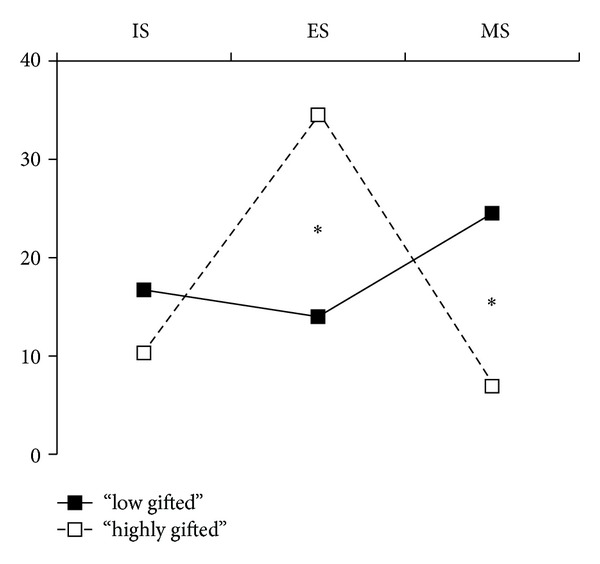
Proportions (%) of “low gifted” (*n* = 114) and “highly gifted” (*n* = 29) children whose scores exceeded norms on “Internalized problems,” “externalized problems,” or both. *P < 0.05. IS: internalized syndrome; ES: externalized syndrome; MS: mixed syndrome.

Mean CBCL raw scores and subscores across “low gifted” children and “highly gifted” children are listed in Table 3. Mean SC subscore was significantly higher in low gifted children (3.0 ± 2.8  *versus *1.7 ± 1.7; *P* < 0.05); other comparisons showed no significant difference. Proportions of low gifted and highly gifted children with internalizing, externalizing, or mixed syndromes are depicted in Figure 1. Proportion was significantly higher in the highly gifted subgroup for the externalizing syndrome (34.5% *versus* 14.0%;  *P* < 0.05) and in the low gifted subgroup for the mixed syndrome (24.5% *versus* 6.9%; *P* < 0.05); there was no significant difference regarding the internalizing syndrome (16.7% in the low gifted subgroup *versus* 10.3%).

**Figure 2:**
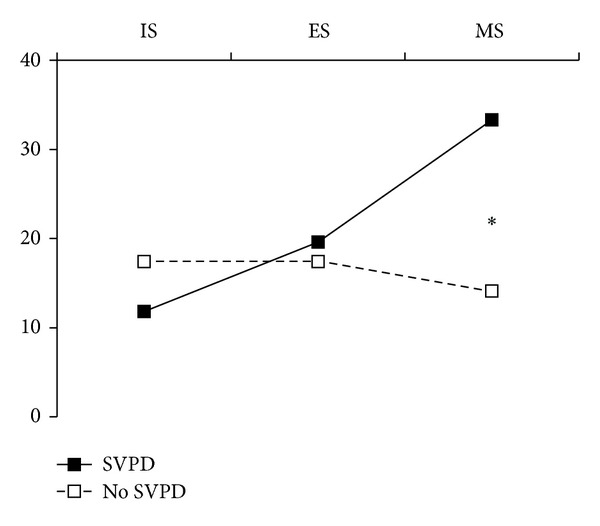
Proportions (%) of gifted children with (*n* = 51) and without (*n* = 92) a significant verbal-performance discrepancy (SVPD) whose scores exceeded norms on “internalized problems,” “externalized problems,” or both. *P < 0.01. IS: internalized syndrome; ES: externalized syndrome; MS: mixed syndrome.

Mean CBCL raw scores and subscores across gifted children with and without an SVPD are listed in Table 4. Children with a significant SVPD scored significantly higher on the EP score (mean: 18.2 ± 8.8  *versus*  14.8 ± 9.9;  *P* < 0.05) and on the AB subscore (14.3 ± 6.5  *versus*  11.5 ± 7.7; *P* < 0.05). Proportions of gifted children with and without an SVPD who displayed internalizing, externalizing, or mixed syndromes are depicted in Figure 2. Proportion was significantly higher in the SVPD subgroup for the mixed syndrome (33.3% *versus* 19.1%; *P* < 0.01); other comparisons showed no significant difference (internalizing syndrome: 11.8% in the SVPD subgroup *versus* 17.4%; externalizing syndrome: 19.6% *versus* 17.4%).

## 4. Discussion

Our results show that clinically referred gifted children display significant and varied behavioral problems, which confirms our first hypothesis. Taking main French CBCL surveys as references [31, 32], results observed in our gifted group are situated between those obtained in the general population and those obtained in psychiatric outpatient clinics, but closer to the latter. This indicates that clinically referred gifted children represent overall a behaviorally pathological group, of rather moderate symptomatic intensity.

This conclusion could appear contradictory with the fact that no gifted child had a mental disorder according to DSM-IV-TR. However, It must be stressed in this respect that the CBCL model, which provides an empirically based dimensional approach of childhood behavioral, emotional, and social problems, has been devised precisely as a complement to categorical nosology in child and adolescent psychiatry [35] in order to compensate some of its intrinsic limits [12, 36]. Indeed, it is well established that a significant number of clinically referred children with behavioral problems do not enter any diagnostic category in DSM-IV-TR [36, 37], whereas a significant proportion of them display discriminating CBCL profiles [12, 35]. Incidentally, this led international experts to consider introducing new diagnostic categories when designing the recently published fifth version of the *Diagnostic and Statistical Manual of Mental Disorders*—for example, the very debated “severe mood dysregulation disorder” or “disruptive mood dysregulation disorder” categories [38]—which justifications were to reflect the conditions of these “nosologic orphans” [39]. Most of the gifted children in this study, who displayed distributed behavioural profiles, could belong to this still imprecise but symptomatically significant categories.

This is consistent with the result that, contrary to the second hypothesis, internalizing behavioral problems did not predominate over externalizing ones in the gifted children. This illustrates the dispersion of individual behavioral profiles and the fact that many children displayed predominantly externalizing behavioral problems or a mixed pattern of both internalizing and externalizing problems. Epidemiological literature showed a gradient of severity in the symptomatic nature of behavioral syndromes, internalizing syndromes being associated with better clinical outcomes than externalizing syndromes [40, 41] and both with better outcomes than mixed syndromes [41, 42]. Thus, even if our clinically referred gifted children group globally displayed behavioural problems in the low symptomatic range, the symptomatic nature of behavioral syndromes was more severe than expected.

We found that very high IQs among the gifted were not at all associated with increased behavioral problems, which does not support the third hypothesis. On the contrary, it was low gifted children who displayed more somatic complaints. Also, children with very high IQs displayed less mixed syndromes than low gifted. All these findings do not corroborate the usual claim that children with higher IQs among gifted are more behaviorally impaired than others. In contrast, and supporting our fourth hypothesis, gifted children with an SVPD exhibited psychopathology of relatively severe nature implying emotional and behavioral dysregulation.

In the field of psychopathology, SVPD is a classical feature of Asperger syndrome [43], a mild form of pervasive developmental disorder (PDD) with which behaviourally impaired children with intellectual giftedness often share characteristics [44]: verbal precocity, hyperlexia, hypercalculia, semantic hypermnesia, absorbing interests in specialized topics (with limited social sharing), social withdrawal, anxiety, excessive perfectionism, perceptive hypersensitivity, and motor clumsiness. Intellectual giftedness is common in mild forms of PDDs [45, 46], where this cooccurrence has been conceptualized as one of “twice-exceptionalities” [46]. These children with PDDs and intellectual giftedness exhibit both internalizing and externalizing behavioral problems [46]. As PDDs are thought to represent the high-level cooccurrence of continuously distributed quantitative traits [47], it could be hypothesized that a significant proportion of clinically referred gifted children may be situated at the border of such developmental atypicalities. Incidentally, it has been observed that gifted children with behavioral impairment tend to minimize their problems [48, 49], which could reflect defective coping implying denial [50] and thus corroborate the hypothesis that they globally display psychopathological features of rather severe symptomatic nature.

Several limitations must be acknowledged when interpreting results of this study. The first one is the definition of giftedness on the single basis of high IQ. Indeed, giftedness has been conceptualized as additionally entailing increased creativity [51], and it is possible that not all children in this study would have remained labeled as gifted using such a restrictive definition. However, the definition which was used here was the minimal and most consensual one [1]. Secondly, while the gifted and normative groups were matched for sex, thus allowing a control for boys' overrepresentation in the former—which is also found in the general population of gifted [52]—when comparing both, we did not control the effect of parental high academic levels—which is another long known feature of gifted children [15]—on behavioral profiles. However, since our first hypothesis was only descriptive, statistical control of socioeconomic variables was unneeded; incidentally, considering that high academic levels of parents are associated with lower child behavioral problems [53], such a statistical control would probably have amplified contrasts between the two groups. A third limitation is the absence in the gifted group of children with an FSIQ higher than 160, which restricts our testing of the third hypothesis. Actually, such children are very few (approximately 1/10000 in the general population; [1]), but their total absence in our clinical cohort suggests that they are not the most behaviorally impaired across the gifted IQ range. Finally, whereas SVPD is a well-established indicator of cognitive imbalance [54], it would be useful in future research to characterize developmental asynchrony more precisely, for example, with Piagetian concrete and formal operational tasks [55], whose combination with IQ tests allows a deeper description of reasoning heterogeneity [56].

To conclude, results of this study suggest that developmental asynchrony matters when considering psychopathology in gifted children. Further research would be needed in order to clarify the psychopathological vulnerabilities of gifted children and their clinical expressions.

## Figures and Tables

**Table 1:** Sociodemographic characteristics of gifted and control groups.

	Gifted	Controls
	*n* = 143	
Sibling rank		
1st	76 (53.1%)	67 (46.9%)
2nd	38 (26.6%)	54 (37.8%)
3rd or more	29 (20.3%)	22 (15.4%)
Matrimonial situation		
In couple	136 (95.1%)	133 (93.0%)
Single	7 (4.9%)	10 (7.0%)
Parent employed		
Father	132 (92.3%)	137 (95.8%)
Mother	97 (67.8%)	109 (76.2%)
Parent with high educational level		
Father*	114 (79.7%)	81 (56.6%)
Mother*	110 (76.9%)	82 (57.3%)

*P < 0.001.

**Table 2:** Mean CBCL scores and subscores across “gifted” and “control” groups. All comparisons showed significant differences for *P* < 0.001.

	Gifted	Controls
	*n* = 143	
TS	44.0 ± 21.3	24.7 ± 17.1
IP	16.0 ± 8.8	9.2 ± 7.2
EP	16.0 ± 9.6	8.6 ± 6.9
WI	4.2 ± 2.7	2.7 ± 2.3
SC	2.8 ± 2.7	1.5 ± 2.1
AD	9.0 ± 5.7	5.0 ± 4.5
SP	3.9 ± 3.0	2.1 ± 2.4
TP	1.5 ± 1.9	0.5 ± 0.9
AP	6.6 ± 4.1	4.3 ± 3.6
DB	3.4 ± 2.4	1.7 ± 1.6
AB	12.5 ± 7.4	5.9 ± 6.9

TS: total score; IP: internalized problems; EP: externalized problems; WI: withdrawn; SC: somatic complaints; AD: anxious/depressed; SP: social problems; TP: thought problems; AP: attention problems; DB: delinquent behaviour; AB: aggressive behaviour.

**Table 3:** Mean CBCL raw scores and subscores across “low gifted” children and “highly gifted” children.

	Low gifted	Highly gifted
	*n* = 114	*n* = 29
TS	44.8 ± 21.6	41.1 ± 20.4
IP	16.7 ± 8.9	13.3 ± 7.6
EP	16.0 ± 9.9	16.1 ± 8.4
WI	4.4 ± 2.7	3.8 ± 2.9
SC*	3.0 ± 2.8	1.7 ± 1.7
AD	9.3 ± 5.8	7.8 ± 5.3
SP	3.9 ± 2.9	4.1 ± 3.3
TP	1.6 ± 1.9	1.3 ± 1.8
AP	6.7 ± 4.2	6.2 ± 4.0
DB	3.5 ± 2.5	3.2 ± 2.2
AB	12.4 ± 7.5	12.9 ± 6.8

*P < 0.05.

TS: total score; IP: internalized problems; EP: externalized problems; WI: withdrawn; SC: somatic complaints; AD: anxious/depressed; SP: social problems; TP: thought problems; AP: attention problems; DB: delinquent behaviour; AB: aggressive behaviour.

**Table 4:** Mean CBCL raw scores and subscores across gifted children with and without a significant verbal-performance discrepancy (SVPD).

	SVPD	No SVPD
	*n* = 51	*n* = 92
TS	48.4 ± 20.0	41.6 ± 21.8
IP	17.7 ± 9.5	15.0 ± 8.2
EP*	18.2 ± 8.8	14.8 ± 9.9
WI	4.5 ± 2.8	4.1 ± 2.7
SC	3.2 ± 3.5	2.5 ± 2.1
AD	10.0 ± 5.8	8.4 ± 5.6
SP	4.2 ± 2.8	3.8 ± 3.0
TP	1.5 ± 1.8	1.6 ± 1.9
AP	7.1 ± 3.9	6.4 ± 4.3
DB	3.7 ± 2.5	3.3 ± 2.4
AB*	14.3 ± 6.5	11.5 ± 7.7

*P < 0.05.

TS: total score; IP: internalized problems; EP: externalized problems; WI: withdrawn; SC: somatic complaints; AD: anxious/depressed; SP: social problems; TP: thought problems; AP: attention problems; DB: delinquent behaviour; AB: aggressive behaviour.

## Abbreviations

AB: Aggressive behavior
AD: Anxious/depressed
AP: Attention problems
CBCL: Child Behavior Checklist
DB: Delinquent behavior
DSM-IV-TR: Fourth version revised of the *Diagnostic and Statistical Manual of Mental Disorders *
EP: Externalizing problems
FSIQ: Full-scale intellectual quotient
IP: Internalizing problems
IQ: Intellectual quotient
PDD: Pervasive developmental disorder
SC: Somatic complaints
SP: Social problems
SVPD: Significant verbal-performance discrepancy
TP: Thought problems
TS: Total score
WI: Withdrawn.
